# Bacterial diversity in the rhizosphere of maize and the surrounding carbonate-rich bulk soil

**DOI:** 10.1111/j.1751-7915.2012.00358.x

**Published:** 2012-08-06

**Authors:** Adela García-Salamanca, M Antonia Molina-Henares, Pieter van Dillewijn, Jennifer Solano, Paloma Pizarro-Tobías, Amalia Roca, Estrella Duque, Juan L Ramos

**Affiliations:** 1 Department of Environmental Protection, Estación Experimental del Zaidín, Consejo Superior de Investigaciones Científicas18008, Granada, Spain; 2 Bio-Iliberis Research and Development, I +D Department18210, Peligros, Granada, Spain

## Abstract

Maize represents one of the main cultivar for food and energy and crop yields are influenced by soil physicochemical and climatic conditions. To study how maize plants influence soil microbes we have examined microbial communities that colonize maize plants grown in carbonate-rich soil (pH 8.5) using culture-independent, PCR-based methods. We observed a low proportion of unclassified bacteria in this soil whether it was planted or unplanted. Our results indicate that a higher complexity of the bacterial community is present in bulk soil with microbes from nine phyla, while in the rhizosphere microbes from only six phyla were found. The predominant microbes in bulk soil were bacteria of the phyla *Acidobacteria*, *Bacteroidetes* and *Proteobacteria*, while *Gammaproteobacteria* of the genera *Pseudomonas* and *Lysobacter* were the predominant in the rhizosphere. As *Gammaproteobacteria* respond chemotactically to exudates and are efficient in the utilization of plants exudate products, microbial communities associated to the rhizosphere seem to be plant-driven. It should be noted that *Gammaproteobacteria* made available inorganic nutrients to the plants favouring plant growth and then the benefit of the interaction is common.

## Introduction

The taxonomical and functional structures of soil microbial communities are influenced by biotic and abiotic factors including the physicochemical characteristics of soil itself, water availability, climate conditions, presence of plants, plant types, and the interactions with other soil prokaryotic and with lower or higher eukaryotic organisms (Pennanen *et al*., 1999; Oline, 2006; Jones *et al*., 2009). Plants exert selective pressure on soil microbial populations through modification of the physicochemical characteristics of the surrounding soil and the excretion of exudates consisting of amino acids and organic acids, proteins and other chemicals that act as chemoattractant or repellent molecules (Rambelli, 1973; Espinosa-Urgel *et al*., 2002; Shaw *et al*., 2006; Acosta-Martínez *et al*., 2008; Haichar *et al*., 2008; Berg and Smalla, 2009; DeAngelis *et al*., 2009; Lacal *et al*., 2011). In this study we have focused our attention on the influence of maize, one of the main plant cultivars for animal and human foodstuff. It is known that maize seeds exude a large variety of amino acids, sugars and some weak organic acids that modify the surrounding soil (Vílchez *et al*., 2000) and that the continuous supply of nutrients via root exudates allows the establishment of a dynamic and nutrient-rich niche in the rhizosphere where the total number of microbes is higher than in bulk soil (Kowalchuk *et al*., 2002; Nunes da Rocha *et al*., 2009). Bacteria that colonize the roots and surrounding soil can be pathogens, saprophytes or beneficial plant growth promoters. Among plant growth promoting rhizobacteria (PGPR) are those that solubilize phosphate and nitrogen (Cocking, 2003; Rodríguez *et al*., 2006; Matilla *et al*., 2007), and that protect plants against pathogens via the production of antibiotics, antifungal chemicals and insecticides (Preston *et al*., 2001; Berg *et al*., 2005).

It is known that only a fraction of soil microbes can be cultured. Because of this limitation a variety of fingerprinting methods, dependent or independent of cloning-sequencing procedures, have been developed (Fierer and Jackson, 2006; Smalla *et al*., 2007). Microbial phylogenetic diversity can be defined by analysing the gene sequences encoding 16S rRNAs isolated from environmental samples (Giovannoni *et al*., 1990; DeLong, 1992; Pace, 1997; Huber *et al*., 2002; Hewson *et al*., 2003; Rappé and Giovannoni, 2003). The resulting sequences can then be used to generate taxonomic inventories of microbial populations, and the abundance curves from observed frequencies of sequences can be used to predict the number of different microbial taxa in a specific sample (Chao, 1984; Chao *et al*., 1992; Curtis *et al*., 2002). Therefore, 16S rRNA analysis is considered an effective tool to compare bacterial community patterns from different samples collected from different environments (Kowalchuk *et al*., 2002; Smalla *et al*., 2007; Haichar *et al*., 2008).

The present study was aimed to examine how maize plants influence the diversity of microbial communities in a typical carbonate-rich Mediterranean soil. Maize is used as a model plant in this study because of its agronomical importance and its use in soils with a wide range of pHs. In this study we have concentrated on a relatively high pH carbonate-rich soil typical of the South Spain (Table 1). We have examined bacterial diversity in the rhizosphere (soil attached to roots) and bulk soil using culture-independent PCR-based methods. Our findings show that plants exerted selective pressure on the microbial communities, causing enrichment of *Gammaproteobacteria* in the rhizosphere, a group of microbes that are chemotactically attracted by maize exudates that are rich in energy sources.

**Table 1:** Physicochemical properties of soils used in this study to grow maize

Test description	
Active lime	3.70%
Carbonates	13.6%
Classification	Type clay loam
Assimilable phosphorus	11 ppm
Humic matter	0.79%
Total nitrogen	0.072%
pH	8.5
Assimilable potassium	205 ppm
Salinity pretest	0.17 mmhos cm^−1^
Clay texture	31.30%
Sand texture	37.02%
Silt texture	31.68%

Soil assays were performed by the Andalucian Service of soil analysis laboratory using International Standard methods.

## Results and discussion

**Fig. 1:**
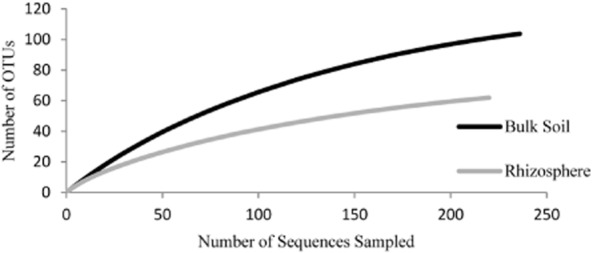
Rarefaction analysis for rhizosphere and bulk soil. Rarefaction curves were constructed using DOTUR software. Rarefaction is shown for OTU with differences that do not exceed 3%.

Roots progressing in ‘bulk soil’ introduce labile carbon and nutrients while creating water ways and deposits of antimicrobial compounds and hormones (Brimecombe *et al*., 2001; Bringhurst *et al*., 2001; Hawkes *et al*., 2007) in time (hours or days) (Lubeck *et al*., 2000). As many soil microbes exhibit limitations to carbon (Paul and Clark, 1996), they could be expected to respond quickly to root-induced changes, by reprogramming their activity (Heijnen *et al*., 1995; Herman *et al*., 2006). We have analysed microbial biodiversity in bulk soil, as well as in the more tightly root-adhering soil as is the rhizosphere of maize plants. To this end the different types of soil were collected and total DNA extracted and used for a PCR-based 16S rDNA gene diversity survey of microbial communities (see *Experimental procedures*). Species richness was represented in rarefaction curves and was measured based on at least 220 sequences and the number of operational taxonomic units (OTUs) using a cut-off of 97% for sequence similarity, a commonly known level for comparative analysis of whole and partial 16S rRNA sequences (Konstantinidis *et al*., 2006). Rarefaction analysis was used to compare bacterial richness between the rhizosphere soil and bulk soil samples. Figure 1 is a rarefaction curve based on best match for each sequence of 16S rDNA genes and their frequency of recovery. The results show that as the number of sequences in the samples increased, the number of OTUs tended to level (Fig. 1, Fig. S1 for cut-off values different of 97% sequence similarity). The numbers of OTUs for a similar number of sequences were always higher in the bulk soil than in the rhizosphere.

A series of statistical analyses were performed and several indexes related to biodiversity were calculated to estimate the biodiversity of samples (Table 2). While the Chao 1 index suggested that the maximum OTU value for bulk soil and rhizosphere should be 118 and 78, Good's coverage index gave 0.57 for bulk soil and 0.73 for the rhizosphere (Good, 1953; Zaballos *et al*., 2006). The Shannon index value was 4.40 for bulk soil and 3.42 for the rhizosphere, while the Simpson's index value was 0.01 for bulk soil and 0.059 for the rhizosphere. These results suggest that the bacterial community present in bulk soil seems more complex than that of the rhizosphere, although we consider that our analysis may underestimate the true richness of it because of the limited number of sequences we obtained, although the Chao 1 value versus the OTU coverage indicates that our analysis had sufficient depth.

**Table 2:** Statistical indexes

	Bulk soil	Rhizosphere
Good index	0.57	0.73
Shannon index	4.4	3.42
Simpson index	0.01	0.059
Chao 1	118	78

DOTUR software was used to compute the statistical indexes for the bacterial sequences.

Phylogenetic reconstruction showed that the sequences were unevenly scattered through the phylogenetic tree (see Figs S2–S4). In the rhizosphere niche six phyla groups were recovered, whereas nine phyla were recovered from bulk soils (Table 3). 16S rDNA gene sequences in the bulk soil belonged predominantly to three phyla including *Acidobacteria* (∼39%), *Bacteroidetes* (∼24%) and *Proteobacteria* (∼20%). Other typical soil microorganisms included *Planctomycetes*, *Actinobacteria* and uncultured members of the TM7 and the OP11 candidate divisions (non-culturable microbes) were found. Similar proportions of these phyla were reported in agricultural and forest soil samples (Roesch *et al*., 2007; Fulthorpe *et al*., 2008; Uroz *et al*., 2010).

**Table 3:** Phylogenetic affiliation of bacterial 16S rRNA genes^a^

Phylogenetic group	Bulk soil (%)	Rhizosphere (%)
Candidate division *OP11*	0.4	0
Candidate division *TM7*	0.8	3.9
*Cyanobacteria*	2.9	0
*Actinobacteria*	2	1.3
*Acidobacteria*	38.8	6.9
*Planctomycetes*	2.1	0
*Deltaproteobacteria*	2.4	0
*Bacteroidetes*	23.7	1.7
*Chloroflexi*	1.2	0
*Alphaproteobacteria*	4.1	13.7
*Betaproteobacteria*	10.2	3
*Gammaproteobacteria*	6.5	65.2
*Firmicutes*		1.7
Unclassified *Bacteria*	4.9	2.6

a Percentage of clones assigned to known and candidate divisions from the 16S rRNA gene libraries from bulk soil and rhizosphere.

In the rhizosphere of *Avena fatua* DeAngelis and colleagues (2009) reported that a significantly larger number of live cells were detected in the rhizosphere in comparison with bulk soil; their study reported as many as 10-fold more cells detected in the root hairs and the root tip rhizosphere in comparison with bulk soil. In that study the authors used ribosomal RNA-targeted oligonucleotide microarrays (Phylochips) and identified the presence of typical rhizosphere phyla such as *Proteobacteria* and *Firmicutes*, as well as other less well-documented rhizosphere colonizers such as *Actinobacteria*, *Verrucomicrobia* and *Nitrospira*. Richness of *Bacteroidetes* and *Actinobacteria* decreased in soil close to the root tip in comparison with bulk soil, but then increased in older root areas.

The rhizosphere soil showed a shift in the most frequently represented microbes and an overall reduction in the number of phyla represented (Table 3). Weisskopf and colleagues (2005) also previously reported a decrease in the richness of bacterial communities from the bulk to the rhizosphere soil, when culturable bacteria were analysed.

The most predominant 16S rRNA gene sequences in the rhizosphere were those of *Gammaproteobacteria* (∼65%) followed by *Alphaproteobacteria* (∼14%) and *Acidobacteria* (∼7%) (Table 3). In a recent meta-analysis of 19 libraries of bacterial clones associated to the roots of 14 plant species, over 1200 distinguishable taxa from 35 different taxonomic orders were described (Hawkes *et al*., 2007). *Proteobacteria* dominated the rhizosphere in 16 of the 19 studies included, presumably because of their relatively rapid growth rates (Atlas and Bartha, 1998). Our observations that *Proteobacteria* are frequent in rhizosphere soils are in agreement with studies carried out with microarrays to detect soil bacteria by Sanguin and colleagues (2006). Our data also showed that the proportion of *Actinobacteria* found in bulk and rhizosphere soil is independent on the presence of plants. This finding is in agreement with the results by Acosta-Martínez and colleagues (2008), who found that levels of *Acidobacteria* were similar regardless of the type of plantation (grass or wheat) and land management practice.

Our overall results are in line with those of Kowalchuk and colleagues (2000), who showed that using culture-independent techniques wild plant species were able to influence the composition of bacterial diversity in the rhizosphere. In their specific study they compared the influence of *Cynoglossum officinale* (hound's tongue) and *Cirsium vulgare* (spear thistle) on soil-borne bacterial communities and found differences in the corresponding microbial communities of the rhizosphere.

The ability of plants to alter microbial diversity and distribution in the rhizosphere may be due to their ability to create a microenvironment that is rich in carbohydrates, carboxylic acids and amino acids, and therefore differences in plant exudates may be behind this discrimination (Grayston *et al*., 1998; Molina *et al*., 2000; Uroz *et al*., 2010). In agreement with the notion that the rhizosphere is more nutrient-rich niche than bulk soil, we found that the levels of alkaline phosphatase, β-glucosidase and dehydrogenase activities in bacterial cells recovered from the rhizosphere were statistically higher than the same activities assayed in cells recovered from bulk soil (Table 4); differences were statistically significant in Student's tests (*P* ≤ 0.05); this increase in activity probably reflected the induction of bacterial catabolic enzymes to nutrients in the exudates, as reported by Vílchez and colleagues (2000), who found a transient increase in proline degradation enzymes in response to maize exudates. Martínez-Iñigo and colleagues (2009) reported that in calcareous soils polluted with heavy metals the microbial enzymatic activity was higher in planted soils than in bare soils at the contamination level of 600 mg of total heavy metals per kilogram of soil. In this soil new bands appeared in the PCR–DGGE profiles of the rhizosphere bacterial community as a response to the exposure to heavy metals, which may indicate that the growth of certain microbes is favoured by the soil/plant interaction. Therefore, soil microorganisms in the rhizosphere show higher levels of activities related to C, N and P cycles, likely representing their induction in response to nutrients. This kind of orchestrated response is known to be under the control of multiple transcriptional regulators (Ishihama, 2010).

**Table 4:** Phosphatase, β-glucosidase and dehydrogenase activities in rhizosphere soil and bulk soil

	Phosphatase	β-Glucosidase	Dehydrogenase
Rhizosphere	325 ± 40	320 ± 50	8 ± 1
Bulk soil	130 ± 15	30 ± 2	1.5 ± 0.3

Enzymatic activities measurements and units are described in *Experimental procedures*. The results are the average of three independent assays performed by duplicate. Data were analysed using STATGRAPHICS Plus Statistical Software (Statistical Graphics, Princeton, NJ, USA) and Student's *t*-test was used to compare mean values.

Previous studies have shown various degrees of a ‘rhizosphere effect’ using either culture-dependent (Miller *et al*., 1989; Germida *et al*., 1998; Grayston *et al*., 1998) or culture-independent strategies (Marilley and Aragno, 1999; Miethling *et al*., 2000; Duineveld *et al*., 2001; Smalla *et al*., 2001; Sanguin *et al*., 2006). The general results of these studies suggest that different plant species differ in the degree and manner in which they influence microbial community structure in the rhizosphere, as was indeed the case when microbial populations of oilseed rape were compared with those of strawberry (Duineveld *et al*., 2001; Smalla *et al*., 2001; Berg *et al*., 2005; Berg and Smalla, 2009). The effect of different plant species on soil microbial communities has been demonstrated for rhizosphere (Grayston *et al*., 1998; Söderberg *et al*., 2002; Iovieno *et al*., 2010) and bulk soil (Myers *et al*., 2001; Carney and Matson, 2006), both for trees (Saetre, 1998; Myers *et al*., 2001; Priha *et al*., 2001; Zak *et al*., 2003; Grayston and Prescott, 2005; Carney and Matson, 2006) and herbaceous plants (Söderberg *et al*., 2002; Zak *et al*., 2003). The influence of plants on the soil microbial community has even been found for different genotypes of the same species (Grayston *et al*., 1998; Schweitzer *et al*., 2008).

In this regard we have carried out detailed analyses of the relative distributions of the genera, families, orders and phyla between microbes in the bulk soil and in the maize rhizosphere (Table 3). These analyses are based on partial 16S rRNA sequence analyses and their location in phylogenetic trees based on the RDP programme (see Figs S2–S4). First, among the genera detected in these two niches only nine common family genera or candidate division were found, namely unclassified *Sphingomonas*, *Acidobacteria* GP6 and GP7, unclassified *Chitinophagaceae*, unclassified *Rhizobiales*, *Pseudomonas*, TM7, and unclassified *Gammaproteobacteria* and *Lysobacter*. Analysis of the eight most abundant genera detected in the bulk soil environment were *Acidobacteria* GP6, GP4 and GP7, *Adheribacter*, *Hymenobacter*, *Massilia*, and unclassified bacteria, each consisting of at least 5% of the total, with GP6 and GP4 being the most abundant (19% and 14% respectively). In the rhizosphere, *Pseudomonas* and *Lysobacter* genera were clearly dominant constituting to 45% of the total microbial abundance, followed by *Pseudoaminobacter*, unclassified *Xanthomonadaceae* and *Acidobacteria* GP7, each in the range of 5–10%.

Analysis of *Proteobacteria* in bulk soil revealed that *Proteobacteria* represent ∼23% of total sequences with *Betaproteobacteria* being the most prevalent (∼45% of total *Proteobacteria*), followed by *gamma* (∼27%), *alpha* (∼18%) and *delta* (11%). Among the *Proteobacteria*, *Burkholderia* was the most common genera followed by *Xanthomonas*. In the rhizosphere, analysis of the *Proteobacteria* phylum showed that there were significantly more *Gammaproteobacteria* (∼75%) than any other *Proteobacteria* with *Pseudomonas* spp. and *Lysobacter* spp. being the dominant genera. This contrasts with studies of the rhizosphere of grape in which there were significantly more *Betaproteobacteria* in the rhizosphere than in the bulk soil, and significantly more *Alphaproteobacteria* in the bulk soil than in rhizosphere (Sanguin *et al*., 2006; Haichar *et al*., 2008).

Bacterial communities are acknowledged as one of the major components of soil function, playing a key role in niche maintenance. Our study shows an increase in the proportion of *Pseudomonas* spp. and *Lysobacter* spp. in the rhizosphere. *Pseudomonas* spp. are well known root colonizers (Molina *et al*., 2000) and are able to proliferate by using plant-secreted amino acids such as proline, lysine, phenylalanine, glutamate and others (Vílchez *et al*., 2000; Espinosa-Urgel and Ramos, 2001; Herrera and Ramos, 2007). In addition, bacteria of this genus exhibit positive chemotaxis towards plant exudates (Espinosa-Urgel *et al*., 2002), a response in which several chemosensors such as McpS are involved (Lacal *et al*., 2011). Because of the parallel increase in the proportion of *Lysobacter* spp. and *Pseudomonas* spp. in the rhizosphere, we suggest that *Lysobacter* spp. could be both able to efficiently use the same carbon and nitrogen sources as *Pseudomonas* spp. and that bacteria of this genera are efficient colonizers of the rhizosphere of plants; however, this will need further *in vitro* assays with culturable *Lysobacter* spp. Our results showed that nitrogen-fixing microbes are of low abundance in this soil and do not apparently play a key role in the mobilization of nitrogen between rhizosphere and bulk soil; instead, microbes capable of metabolizing inorganic nitrogen are present, which is consistent with the historical use of inorganic nitrogen sources at this field site.

In short, our results suggest that the predominant bacterial populations in a carbonate-rich soil are influenced by plants and that this effect is most notable in the rhizosphere, defined here as the root surface and adhering soil. In our study we have analysed 16S rDNA gene sequences, and only assessed the detection of numerically predominant bacterial populations with *Pseudomonas* spp. and *Lysobacter* spp. as the dominant ones. Our results provide data on how certain bacterial populations become dominant in the rhizosphere through a mechanism that is most likely due to the microenvironment created by the presence of maize exudates and bacterial chemotaxis towards nutrients in the exudates. In general, this main conclusion in a soil with a relatively high pH is in agreement with studies that suggest that soil characteristics may be most important factor determining the dominant bacterial populations in bulk soil (Felske and Akkermans, 1998; Kowalchuk *et al*., 2000), while the microbial communities found in the rhizosphere are, to a greater extent, plant-driven.

## Experimental procedures

### Isolation of DNA from soil and rhizosphere samples

Five 1 kg pots were filled with soil collected at the Estación Experimental del Zaidín (Granada), [+37°9′56.50″N, −3°35′31.13″O] 678 m, and each planted with maize seeds. Plants were kept in a greenhouse with 12 h/12 h light–dark cycle, 50% humidity and watered daily. The soil physicochemical parameters were analysed at the ‘Instituto Agroalimentario de Atarfe’ (Table 1). Thirty corn seeds were surface sterilized according to Espinosa-Urgel and colleagues (2000) and sown in pots containing the soil. After 2 weeks maize plants were removed from the soil and the soil which tightly adhered to roots to the plants was separated using glass beads; this soil was the rhizosphere, whereas the soil that did not adhere was taken as the bulk soil. Bulk and rhizosphere soil samples were sieved through a 4 mm mesh (Molina *et al*., 2000).

Soil samples were processed immediately for DNA extraction. Several methods were used to extract DNA and in terms of quality of DNA we found that the most efficient was that in which total DNA was isolated directly from cells after matrix separation by density gradient centrifugation with Nycodenz (Axis-Shield PoC, Norway), as described by Ferrer and colleagues (2011). DNA was extracted using the GNOME®DNA commercial kit (QBIOgene) and visualized using 0.8% (wt/vol) agarose gel electrophoresis.

### Construction of 16S RNA gene clone libraries, DNA sequencing and sequence analysis

For PCR amplification of the 16S rRNA gene serial dilutions of DNA template were used. An approximately 1450 bp amplification product was obtained using universal primers GM3F (5′-AGAGTTTGATCMTGGC-3′) and GM4R (5′-TACCTTGTTACGACTT-3′). Amplification was carried out in 50 μl reaction volume with 2.5 U recombinant *Taq* DNA polymerase, 25 ng of metagenomic DNA, 250 μM of each of the four deoxynucleotide triphosphates, 1.5 mM MgCl_2_, 200 nM of each primer and the appropriate buffer supplied by the manufacturer (Roche), according to the PCR protocol described by Uroz and colleagues (2010).

PCR amplicons were purified through 0.8% (wt/vol) agarose gels. DNA was excised using a QIAQUICK Gel Extraction Kit (Qiagen, Germany) and this DNA was ligated into the pGEM-T plasmid vector (Promega, Madison, WI, USA), with subsequent transformation into competent cells of *Escherichia coli* DH5α. DNA encoding bacterial 16S rRNA were sequenced using the M13 forward and M13 reverse primers. To minimize the effects of random sequencing errors, sequence chromatograms were manually checked to eliminate ambiguities. On average, this stringent trimming procedure reduced the number of sequences by 20% and the average size of the analysed sequences was about 700 bp.

Preliminary phylogenetic analysis of the 16S rRNA clones was performed using the Classifier tool of the Ribosomal Data Project (Cole *et al*., 2009) (confidence level of 85%). Sequences were checked for possible chimeric origin by using the Ribosomal Database Project's CheckChimera program, which is based on the Pintail algorithm (Ashelford *et al*., 2005). Then, phylogenetic inference was carried out using the ARB software package (Ludwig *et al*., 2004). Sequences were automatically aligned using SINA aligner against SILVA SSURef 100 (Pruesse *et al*., 2007) and LTPs100 (Yarza *et al*., 2008). The alignments were manually inspected to correct inaccurately misplaced bases. Two independent reference phylogenetic trees were reconstructed to improve resolution at lower taxonomic levels – one comprising only members of the phylum *Proteobacteria* and a second one containing the remaining bacterial phyla. The phylogeny was reconstructed with the neighbour-joining algorithm using the Jukes-Cantor correction.

ARB-generated 16S sequence alignments were used to create Jukes-Cantor corrected distance matrices. These matrices were used as input for the DOTUR program (see below, Schloss and Handelsman, 2005).

### Nucleotide sequence accession numbers

The 16S rRNA gene sequences of the samples analysed in this study were deposited at the GenBank under accession numbers JN366808–JN367265.

### Index calculations

The microbial diversity was evaluated using several species-diversity indices (Atlas and Bartha, 1998). The DOTUR software program was used to compute the statistical indexes and to generate rarefaction curves (Heck *et al*., 1975). For both libraries the coverage was estimated using the Good index (Good, 1953), and the diversity was calculated using the Shannon-Weiner and Simpson's indexes (Magurran, 1998). Sequences were grouped at equal or higher than 97% identity as the standard cut-off. In addition, we determined the non-parametric index Chao as an estimator of species richness (Heltsche and Forrester, 1983; Chao, 1984; Colwell and Coddington, 1994).

### Determination of soil enzymatic activities

Dehydrogenase activity was determined by the reduction of 2-*p*-iodo-nitrophenyl-tetrazolium chloride (INT) to iodo-nitrophenyl formazan (INTF) as described by Skujins (1976) and modified by García-Gil and colleagues (2000). Dehydrogenase activity was measured using 1 g of soil, following incubation in the dark with 0.2 ml of 0.4% INT for 20 h at 37°C. The INTF was extracted with a mixture of acetone : tetrachloroethene (1.5:1) by shaking vigorously for 2 min and measuring absorbance at 490 nm in a spectrophotometer. Assays without soil and without INT were carried out simultaneously as controls. Activity is expressed as μg INTF produced g^−1^ dry soil h^−1^.

Phosphatase and β-glucosidase activities were determined using disodium *p*-nitrophenyl phosphate (PNPP, 0.115 M) and *p*-nitrophenyl-β-D-glucopyranoside (PNG, 0.05 M) as substrates respectively. These assays are based on the production and detection of *p*-nitrophenol (PNP). Two millilitres of 0.1 M maleate buffer (pH 6.5 for both phosphatase and β-glucosidase activities) and 0.5 ml of substrate were added to a 0.5 g sample and incubated at 37°C for 2 h. The reaction was determined by adding 0.5 M CaCl_2_ and 2 ml of 0.5 M NaOH and the mixture was centrifuged at 3500 *g* for 10 min. The amount of PNP was determined using a spectrophotometer at 398 nm (Tabatabai and Bremner, 1969). The same procedure was followed for the controls except that the substrate was added to the soil immediately before stopping the reaction. Activity is expressed as μg PNP production g^−1^ dry soil h^−1^.

## Supporting information

**Fig. S1.** Rarefaction analysis for (A) bulk soil and (B) rhizosphere samples based on pairwise distance. Rarefaction is shown for OTUs with differences that do not exceed 3%, 5%, or 10%.

**Fig. S2.** Neighbour-joining tree of proteobacterial SSU rRNA gene sequences from clone libraries established from bulk soil community DNA. Clones sequenced in this work are marked in red.

**Fig. S3.** Neighbour-joining tree of non-proteobacterial SSU rRNA gene sequences from clone libraries established from bulk soil community DNA. Clones sequenced in this work are marked in red.

**Fig. S4.** Neighbour-joining tree of proteobacterial SSU rRNA gene sequences from clone libraries established from rhizosphere community DNA. Clones sequenced in this work are marked in red.
