# Supplementary figures and images for: Bacterial diversity in the rhizosphere of maize and the surrounding carbonate-rich bulk soil
Source: Microb Biotechnol. 2012 Aug 6;6(1):36–44. doi: 10.1111/j.1751-7915.2012.00358.x (PMC3815383; doi:10.1111/j.1751-7915.2012.00358.x)

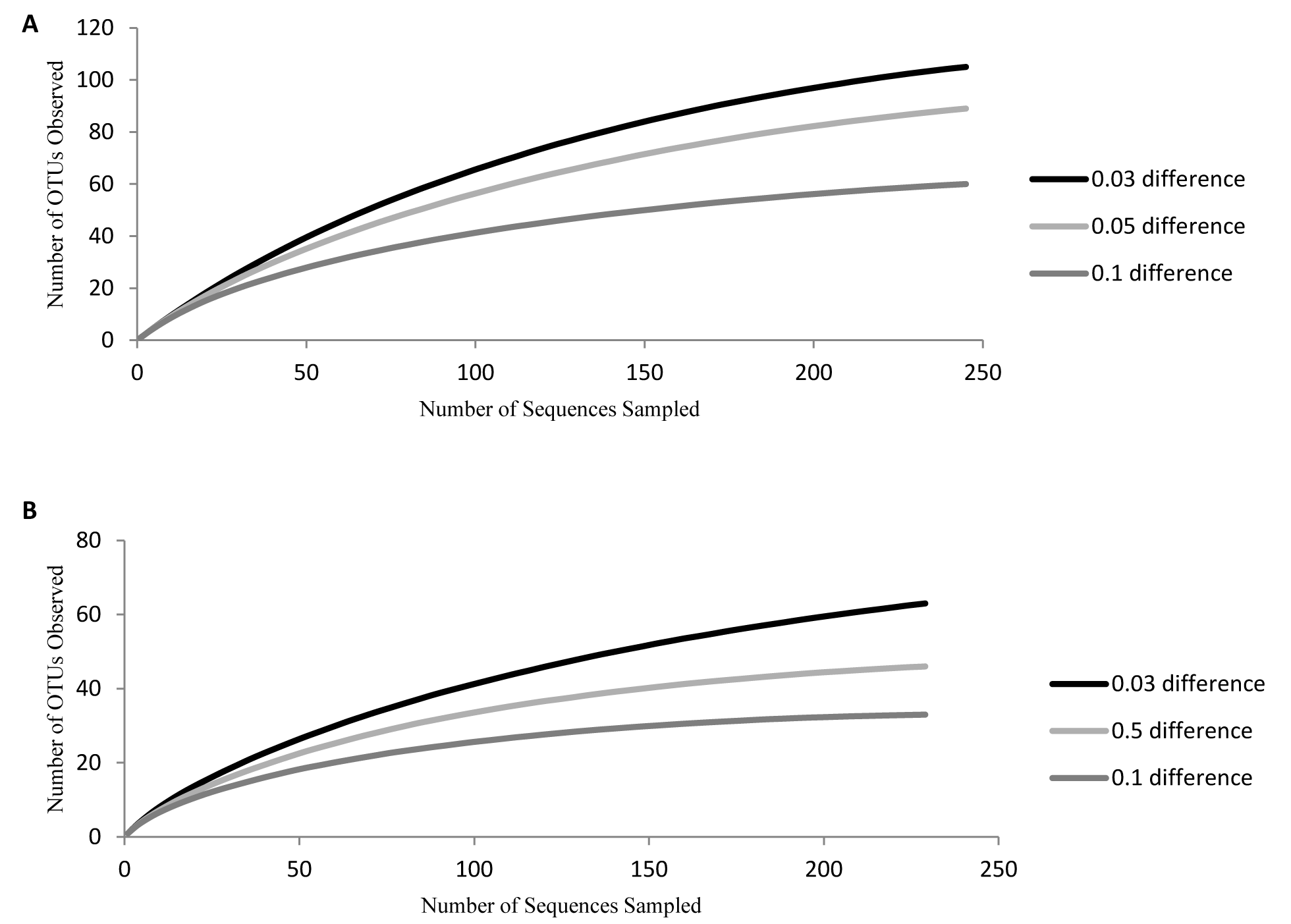

Supplement: Supplementary file 1 [file mbt0006-0036-SD1.tif]

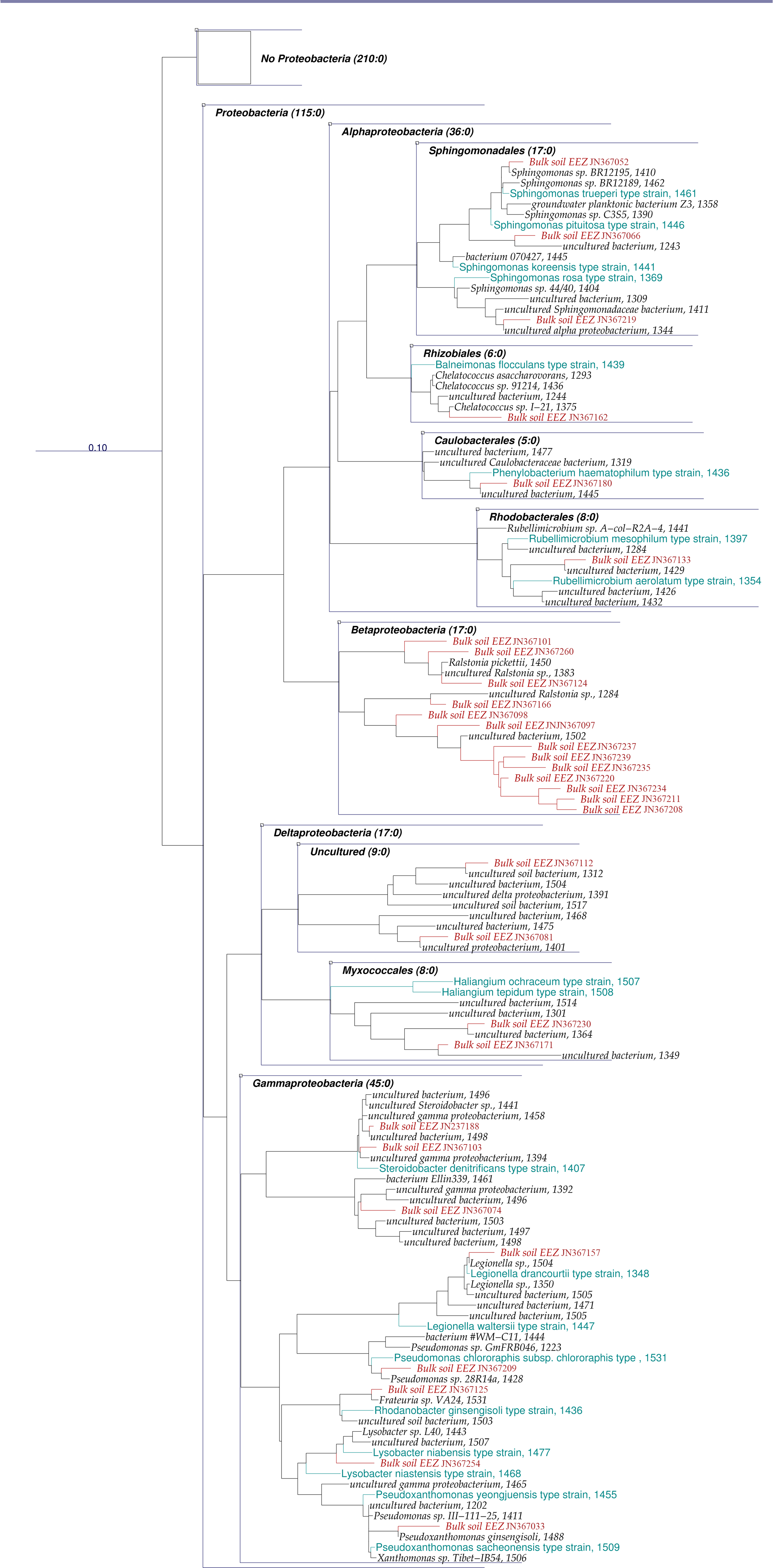

Supplement: Supplementary file 2 [file mbt0006-0036-SD2.tif]

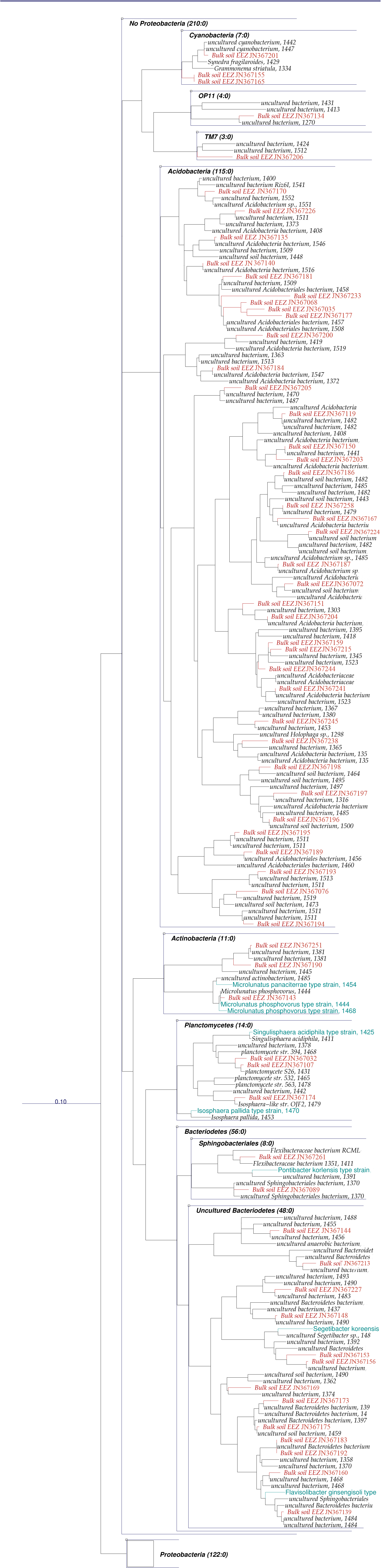

Supplement: Supplementary file 3 [file mbt0006-0036-SD3.tif]

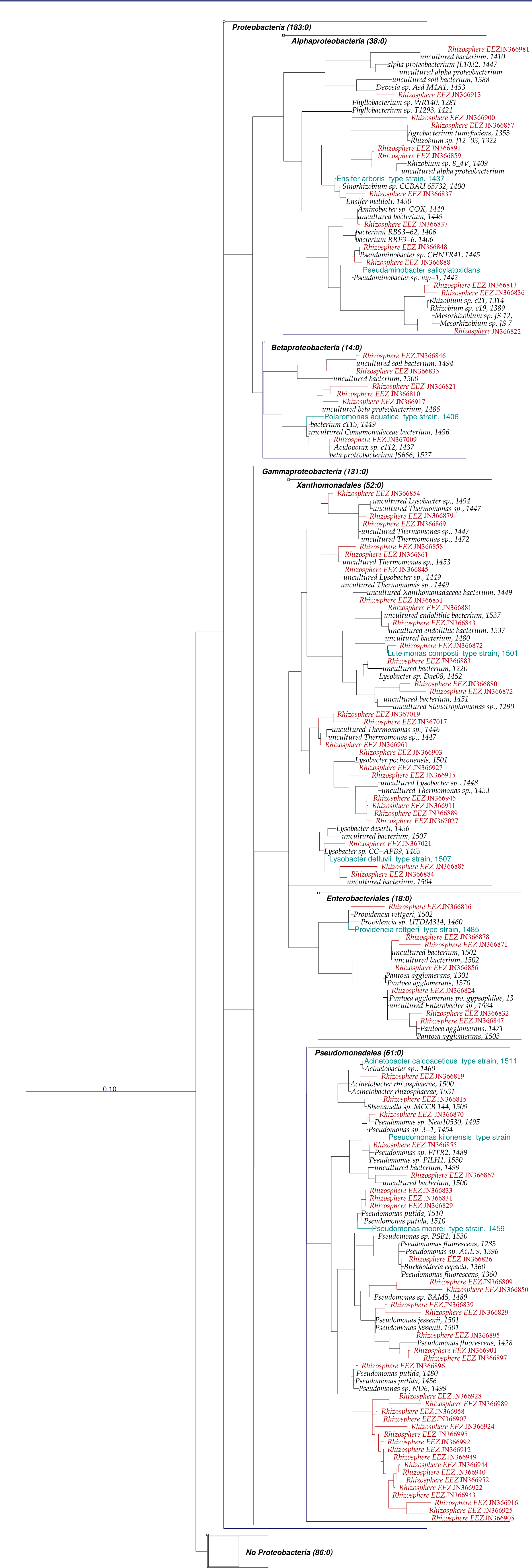

Supplement: Supplementary file 4 [file mbt0006-0036-SD4.tif]
